# Assessment of pathological grade and variants of bladder cancer with a continuous-time random-walk diffusion model

**DOI:** 10.3389/fonc.2024.1431536

**Published:** 2024-08-15

**Authors:** Wei Wang, Jingyun Wu, Qi Shen, Wei Li, Ke Xue, Yuxin Yang, Jianxing Qiu

**Affiliations:** ^1^ Department of Radiology, Peking University First Hospital, Beijing, China; ^2^ Department of Urology, Peking University First Hospital, Institute of Urology, National Research Center for Genitourinary Oncology, Peking University, Beijing, China; ^3^ MR Collaboration, United Imaging Research Institute of Intelligent Imaging, Beijing, China

**Keywords:** urinary bladder neoplasms, pathology, neoplasm grading, tumor microenvironment, diffusion magnetic resonance imaging

## Abstract

**Purpose:**

To evaluate the efficacy of high b-value diffusion-weighted imaging (DWI) with a continuous-time random-walk (CTRW) diffusion model in determining the pathological grade and variant histology (VH) of bladder cancer (BCa).

**Methods:**

A total of 81 patients (median age, 70 years; range, 35-92 years; 18 females; 66 high grades; 30 with VH) with pathologically confirmed bladder urothelial carcinoma were retrospectively enrolled and underwent bladder MRI on a 3.0T MRI scanner. Multi-b-value DWI was performed using 11 b-values. Three CTRW model parameters were obtained: an anomalous diffusion coefficient (D) and two parameters reflecting temporal (α) and spatial (β) diffusion heterogeneity. The apparent diffusion coefficient (ADC) was calculated using b0 and b800. D, α, β, and ADC were statistically compared between high- and low-grade BCa, and between pure urothelial cancer (pUC) and VH. Comparisons were made using the Mann–Whitney U test between different pathological states. Receiver operating characteristic curve analysis was used to assess performance in differentiating the pathological states of BCa.

**Results:**

ADC, D, and α were significantly lower in high-grade BCa compared to low-grade, and in VH compared to pUC (p < 0.001), while β showed no significant differences (p > 0.05). The combination of D and α yielded the best performance for determining BCa grade and VH (area under the curves = 0.913, 0.811), significantly outperforming ADC (area under the curves = 0.823, 0.761).

**Conclusion:**

The CTRW model effectively discriminated pathological grades and variants in BCa, highlighting its potential as a noninvasive diagnostic tool.

## Introduction

The pathological status of bladder cancer (BCa) significantly influences its management strategies. BCa is classified as low- or high-grade based on the extent of nuclear anaplasia and architectural abnormalities (1). Transurethral resection of bladder tumor (TURBT) remains the cornerstone of treatment for low-grade, non-muscle invasive BCa. For lesions assessed as high-grade upon resection, a repeat TURBT is recommended to ensure comprehensive removal (2). High-grade BCa carries an elevated risk for muscle invasion or metastatic disease (3). Predominantly, BCa manifests as pure urothelial carcinoma (pUC); however, up to 25% of cases present morphological features that differ from pUC, including different histologic subtypes and/or divergent differentiations (1, 4), which can be indicative of a more aggressive disease trajectory and adverse outcomes (5, 6). Therefore, accurate evaluation of grade and variant is crucial for tailoring therapeutic strategies and forecasting prognosis for BCa patients.

Identifying BCa pathology relies on invasive TURBT (7). However, insufficient TURBT may lead to underestimation and inaccuracy (8). The apparent diffusion coefficient (ADC), a traditional measurement of diffusion in tissue, has been identified as a promising imaging biomarker for detecting BCa pathology (9). Several studies have reported an inverse correlation between ADC and the grade of BCa (10–13). The underlying mechanism for the correlation of ADC values with pathological characteristics involves microstructural changes within malignancy, including larger cell size and density (9). Besides pathological grade, the importance of reporting variant histology (VH) in BCa has been emphasized in recent years (14, 15). The use of DWI and ADC in evaluating variants is lacking but holds significant potential, as the different histological components within UC may create more complex tissue microenvironments than pUC (16). However, water diffusion in complex tumor tissues exhibits a non-Gaussian distribution, which cannot be simply reflected by ADC from the Gaussian diffusion model (17–19). Therefore, non-Gaussian diffusion models are more applicable for characterizing actual microenvironments within complex structures (20).

Recent preliminary studies have reported that high b-value diffusion-weighted imaging (DWI) based on a non-Gaussian continuous-time random-walk (CTRW) model shows promise in diagnosing and evaluating brain, breast, and liver diseases (21–24). Three diffusion parameters derived from the CTRW model—diffusion coefficient (D), temporal diffusion heterogeneity (α), and spatial diffusion heterogeneity (β)—can quantitatively reflect water molecular diffusion and intravoxel structural heterogeneity (22). The fractional order calculus (FROC) model is a simplification of the CTRW model, focusing only on spatial heterogeneity (25). A recent study demonstrated the utility of the FROC model in grading BCa (19), indicating the potential value of non-Gaussian diffusion models in evaluating BCa pathology. However, the performance of the CTRW diffusion model in assessing BCa pathological status remains unknown and warrants further investigation. This study aimed to investigate the efficacy of quantitative diffusion parameters derived from the CTRW model in characterizing the pathological grade and VH of BCa and to compare it with the conventional mono-exponential model.

## Materials and methods

This retrospective, single-institution study received approval from the hospital’s ethics committee, and the requirement for written informed consent was waived.

### Patient enrollment

A total of 107 patients with suspected bladder tumors, who had not received previous treatment and underwent bladder MRI, were consecutively enrolled from August 2022 to November 2023. Patients were excluded if they met any of the following criteria: (1) absence of pathologically confirmed urothelial carcinoma post-MRI (n = 12); (2) poor image quality for diffusion images (n = 1); or (3) lesion diameter less than 5 mm, precluding reliable analysis (n = 4) (10, 26). Notably, if the tumor could not be entirely resected during TURBT, only a biopsy or partial resection was performed for pathological evaluation at our institution. Consequently, 9 patients were excluded from the study due to incomplete pathological information from TURBT, which did not provide comprehensive data for the entire tumor.

### MRI acquisition

The MR examinations were performed on a 3.0 T MR scanner (uMR 790, United Imaging Healthcare), with patients in the supine position, using a 12-channel body phased-array surface coil. A series of axial diffusion-weighted images were obtained for CTRW model analysis using a single-shot spin-echo echo-planar imaging sequence with the following protocol: repetition time/echo time = 2525 ms/56.9 ms, field of view = 240 × 240 mm², matrix = 96 × 86, slice thickness = 4 mm, intersection gap = 0.4 mm, acquisition time = 7 min, and b-values = 0_1_, 50_1_, 100_1_, 200_1_, 400_1_, 800_2_, 1000_4_, 1500_6_, 2000_8_, 2500_14_, and 3000_16_ s/mm² (the subscript denotes the number of excitations). To ensure adequate bladder distention, patients were instructed to drink 500-1000 mL of water 30 minutes before the scan (27).

### Image analysis

The CTRW model analysis utilized the following formula (23):


(1) $$\begin{array}{c} S=S_{0}E_{\alpha }[-{\left(bD\right)}^{\beta }] \end{array}$$

where S is the signal intensity (SI) at a given b-value, and S_0_ is the SI without diffusion weighting. E_α_ is a Mittag-Leffler function (28), b is the b-value, and D is an anomalous diffusion coefficient typically associated with tissue cellularity. The parameters α and β reflect the intravoxel temporal and spatial diffusion heterogeneity, respectively. Based on **Equation (1)**, three CTRW parameter maps (D, α, and β) were generated pixel-by-pixel by fitting the multi-b-value diffusion imaging data via a Levenberg–Marquardt nonlinear fitting algorithm (21).

The nonlinear fitting proceeded with a segmented approach similar to a previous study (22):

- Estimating D using the DWI with low b-values (b ≤ 1000 s/mm^2^) based on a mono-exponential model.
- Simultaneously estimating α and β using all DWI images while fixing the estimated D for each voxel.

The Mittag-Leffler function was computed using open-source code written by Roberto Garrappa in MATLAB Central (https://www.mathworks.com/matlabcentral/fileexchange/48154-the-mittag-leffler-function), which implemented the optimal parabolic contour (OPC) algorithm (29) and was based on the inversion of the Laplace transform on a parabolic contour suitably chosen in one of the regions of analyticity of the Laplace transform. Its complete form is depicted in the following **Equation (2)**:


(2) $$\begin{array}{c} E_{\alpha }(z)=\sum_{k=0}^{\infty }\frac{z^{k}}{\Gamma (\alpha k+1)} \end{array}$$

The conventional ADC map was calculated using the mono-exponential model formula for comparison:


(3) $$\begin{array}{c} S=S_{0}e^{-bADC} \end{array}$$

In **Equation (3)** S_0_ and S stand for the SI at b values of 0 and 800 s/mm^2^, respectively.

The regions of interest (ROIs) for each patient were manually delineated at the slice with the maximum tumor area on DWI images (b = 800 s/mm²) by two experienced radiologists, with 10 and 13 years of experience in urological radiology, respectively. Necrotic or cystic areas were excluded from the ROIs (25, 30). The mean values of the CTRW parameters (D, α, and β) and ADC were subsequently extracted from the delineated ROIs by the two radiologists through CTRW model fitting. Interobserver agreement was assessed for all diffusion parameters. The average diffusion parameters from the two readers were analyzed and presented in tables and figures.

### Reference standard

The histopathological results from surgeries served as the standard reference for evaluating diagnostic performance. The urologists recorded the location of each transurethral resected tumor using a sector map, which was referenced in the histopathological analysis. All pathological specimens were reviewed by an expert urological pathologist with 15 years of experience. The histopathological information included the histological type, grade, T stage, and histological variants (1).

### Statistical analysis

The analysis was performed using SPSS software (version 26) and MedCalc software (version 20). The Shapiro–Wilk test was employed to assess the normality of data distributions. Continuous variables are presented as mean ± standard deviation or median with interquartile range, depending on the normality test results. Student’s t-test for normally distributed data or a Mann-Whitney U test for non-normally distributed data was used to determine the statistical significance of differences in diffusion parameters between low- and high-grade BCa, and between pUC and VH. The intraclass correlation coefficients (ICCs) of the quantitative diffusion parameters were assessed to evaluate the interobserver agreement between the two radiologists (ICC < 0.20, poor; 0.21-0.40, fair; 0.41-0.60, moderate; 0.61-0.80, good; 0.81-1.00, perfect).

Univariate and multivariate logistic regression analyses were used to select the optimal diffusion parameters and integrate them to establish a predictive model. Receiver operating characteristic (ROC) analysis was used to evaluate the diagnostic performance of individual diffusion parameters and their combination. The area under the ROC curve (AUC) was compared using the DeLong test. The cut-off value was determined based on the Youden index. The sensitivity, specificity, and accuracy were calculated using the maximum Youden index. P values less than 0.05 indicated statistical significance.

## Results

### Patient clinicopathological characteristics

**Figure 1:**
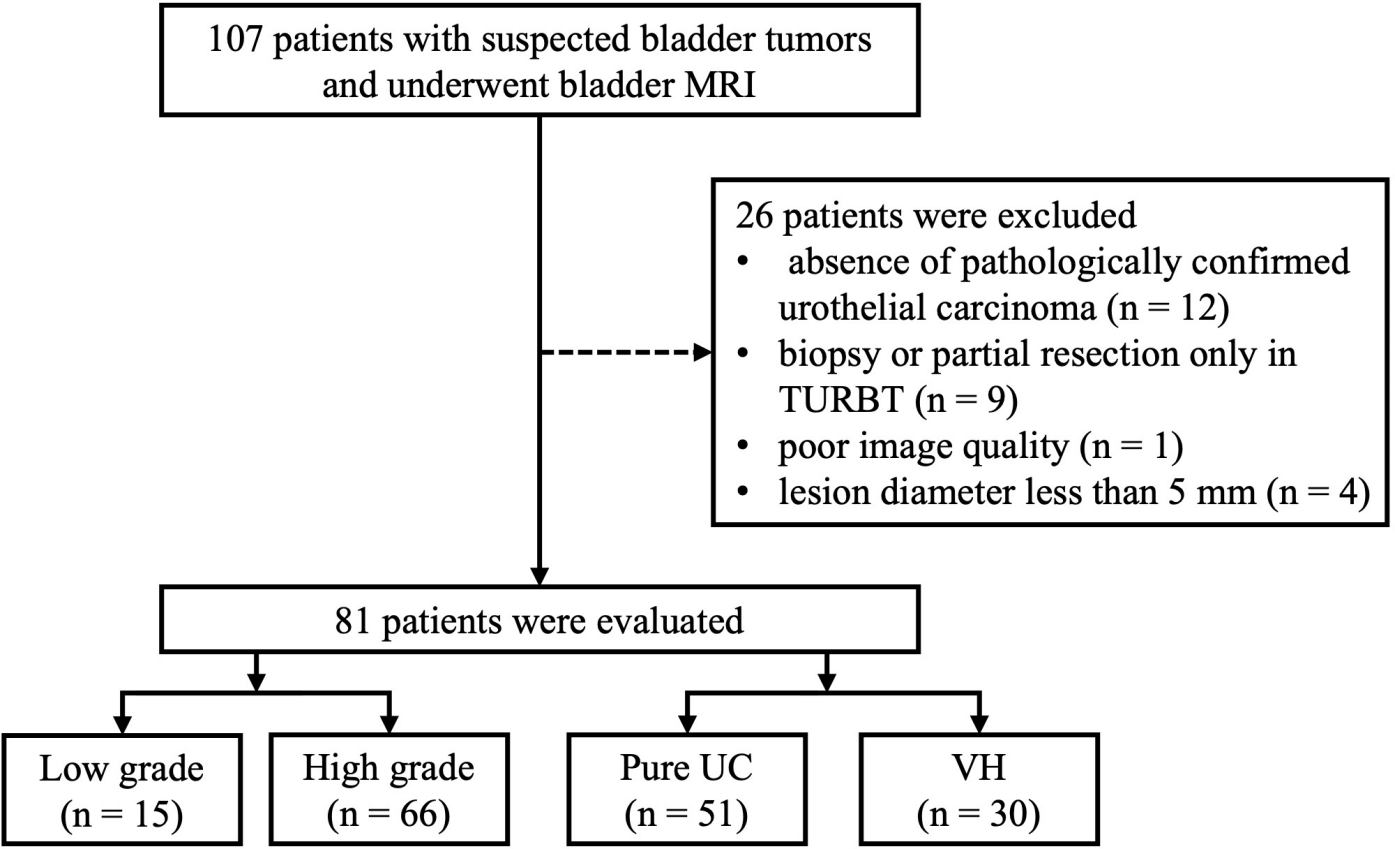
Flowchart of the study. TURBT, transurethral resection of bladder tumor; UC, urothelial carcinoma; VH, variant histology.

A total of 81 patients (18 females; median age, 70 years; interquartile range, 13 years) with 81 lesions (median diameter, 2.4 cm; interquartile range, 2.9 cm) were included in the present study. Twenty-four patients presented with multiple bladder tumors; the largest tumor was designated as the index tumor for subsequent analysis. The study flowchart is shown in **Figure 1**. All patients underwent TURBT after MRI. Among them, 25 patients underwent subsequent cystectomies for further treatment after TURBT. Pathology revealed 43 (53.1%) nonmuscle invasive BCa (4 pTa + 39 pT1) and 38 (46.9%) muscle invasive BCa (16 pT2 + 11 pT3 + 11 pT4). Of these 81 BCa lesions, 15 (18.5%) were low grade, and 66 were high grade. Thirty (37.0%) lesions exhibited other histological types, including 12 squamous, 3 sarcomatoid, 2 nested, 2 glandular, 2 small cell, 2 micropapillary, and 7 mixed (3 glandular + squamous, 2 squamous + sarcomatoid, 1 glandular + sarcomatoid, 1 microcystic + sarcomatoid). The patients’ clinical and pathological information is listed in **Table 1**.

**Table 1:** Clinicopathological characteristics of the patients.

Variables	Characteristics	
Age (years)*	70 (13)	
Gender	Male	63
	Female	18
No. of lesions	Single	57
	Multiple	24
Tumor size (cm)*	2.4 (2.9)	
Pathological T stage	pTa	4
	pT1	39
	pT2	16
	pT3	11
	pT4	11
Pathological grade	Low	15
	High	66
Variant histology	Absence	51
	Presence	30
Surgery	TURBT	56
	Radical cystectomy	25

*Numbers are medians, with interquartile range in parentheses.

TURBT, Transurethral resection of bladder tumor.

### Comparison of ADC and CTRW parameters between different pathological groups

Interobserver agreement for mean ADC, D, α, and β values were excellent, with all the ICCs > 0.8 [ICC (95% confidence interval): ADC, 0.936 (0.903-0.959); D, 0.947 (0.919-0.966); α, 0.900 (0.849-0.935); and β, 0.887 (0.830-0.926)]. The average diffusion MRI metrics measured by the two radiologists were used in the analysis. The detailed values of each diffusion parameter and their differences between the different pathological groups are listed in **Table 2**.

**Table 2:** Comparison of ADC and CTRW parameters between different pathological groups (median, IQR).

Parameter	ADC (×10^-3^ mm^2^/s)	D (×10^-3^ mm^2^/s)	α	β
Low grade	1.421 (0.348)	1.583 (0.682)	0.898 (0.067)	0.870 (0.148)
High grade	1.024 (0.313)	1.021 (0.306)	0.783 (0.099)	0.868 (0.074)
p value	< 0.001*	< 0.001*	< 0.001*	0.808
pUC	1.206 (0.418)	1.257 (0.556)	0.860 (0.141)	0.852 (0.114)
VH	0.953 (0.225)	0.934 (0.179)	0.774 (0.066)	0.893 (0.064)
p value	< 0.001*	< 0.001*	< 0.001*	0.059

*Significant difference with *p* < 0.001.

ADC, apparent diffusion coefficient; CTRW, continuous-time random-walk; IQR, interquartile range; pUC, pure urothelial carcinoma; VH, variant histology.

**Figure 2:**
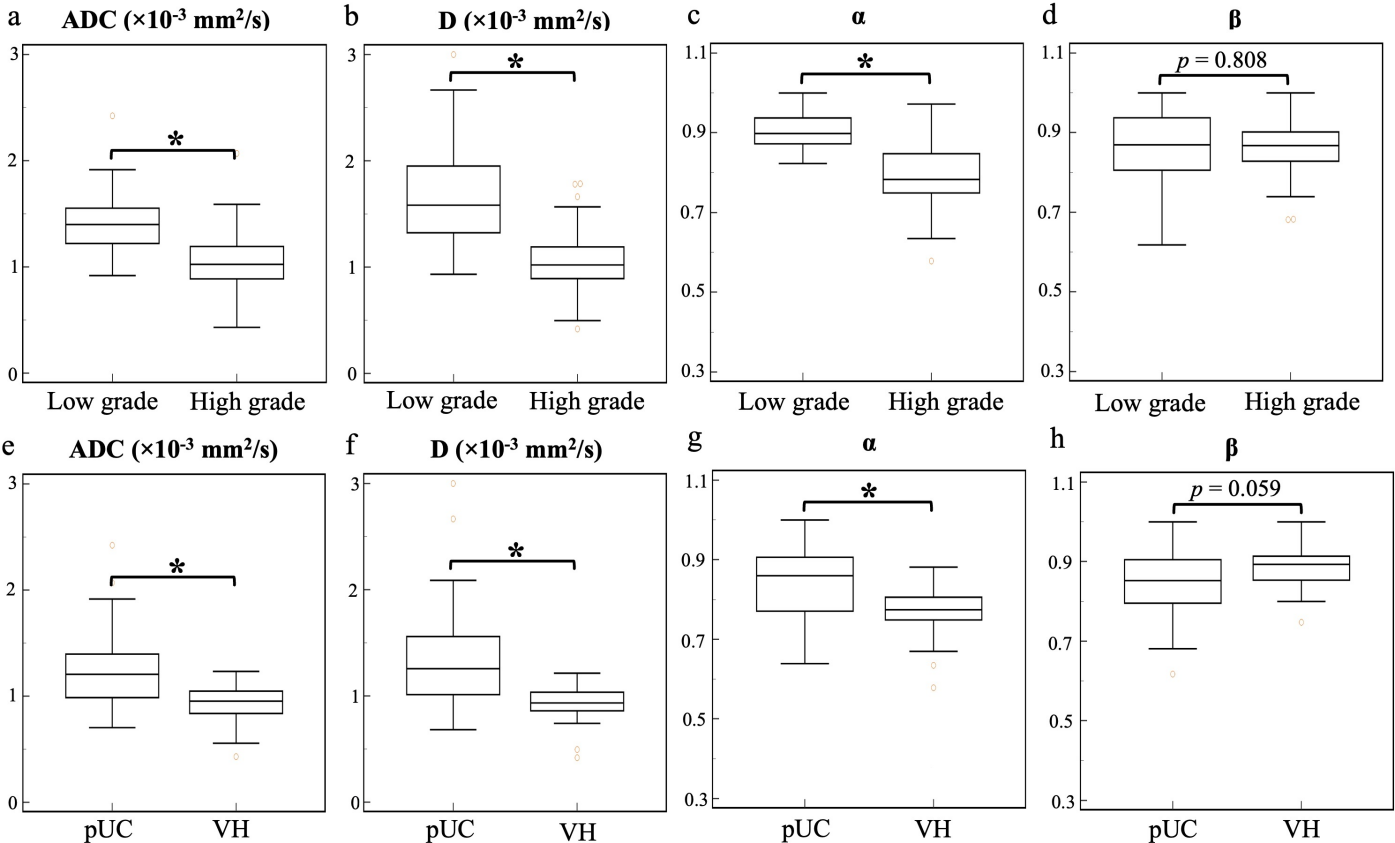
Box-and-whisker plots of the mean apparent diffusion coefficient and continuous-time random-walk parameters (D, α, and β) for low- and high-grade bladder cancer (top row, **A-D**) and pure urothelial carcinoma and variant histology (bottom row, **E-H**). The statistically significant level, *p < 0.001. ADC, apparent diffusion coefficient; pUC, pure urothelial carcinoma; VH, variant histology.

**Figure 3:**
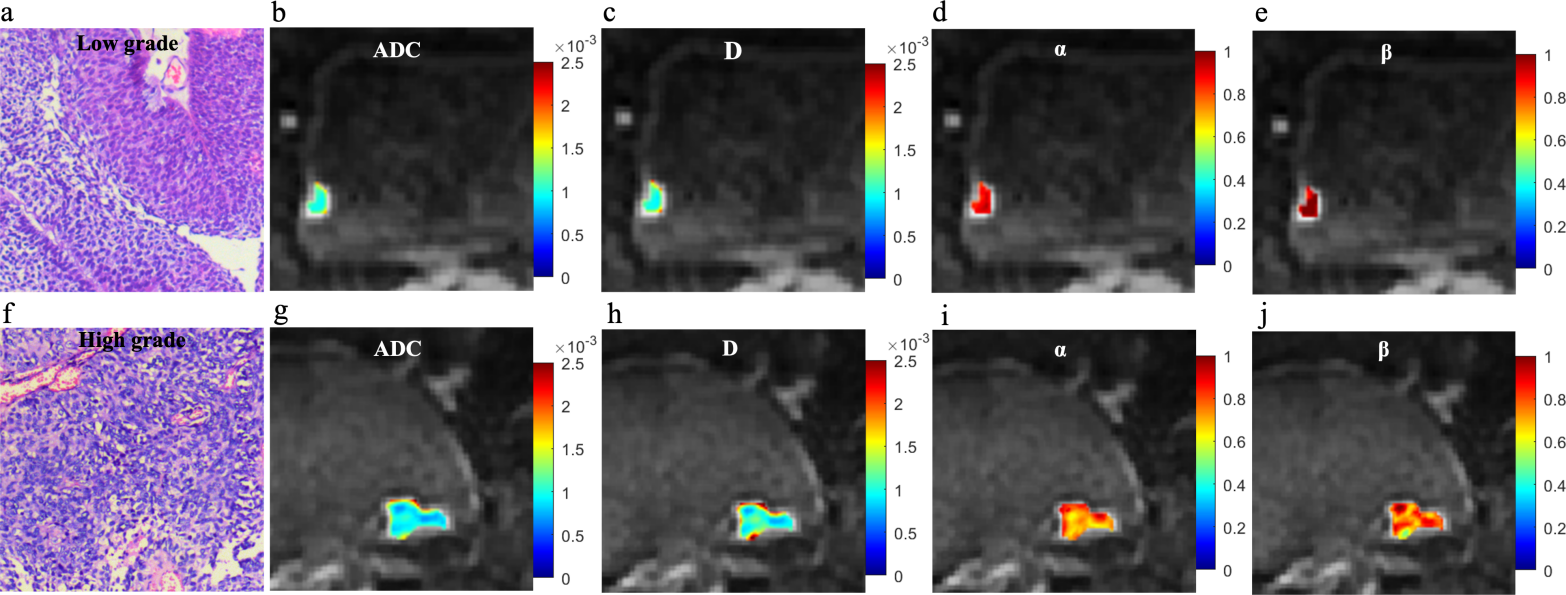
Microscopic pathology and colormaps of the apparent diffusion coefficient and continuous-time random-walk parameters of bladder tumors from a 61-year-old male with low-grade urothelial carcinoma (top row, **A-E**) and an 81-year-old female with high-grade urothelial carcinoma (bottom row, **F-J)**. The mean values of diffusion parameters were lower in high-grade bladder cancer compared to low-grade bladder cancer.

**Figure 4:**
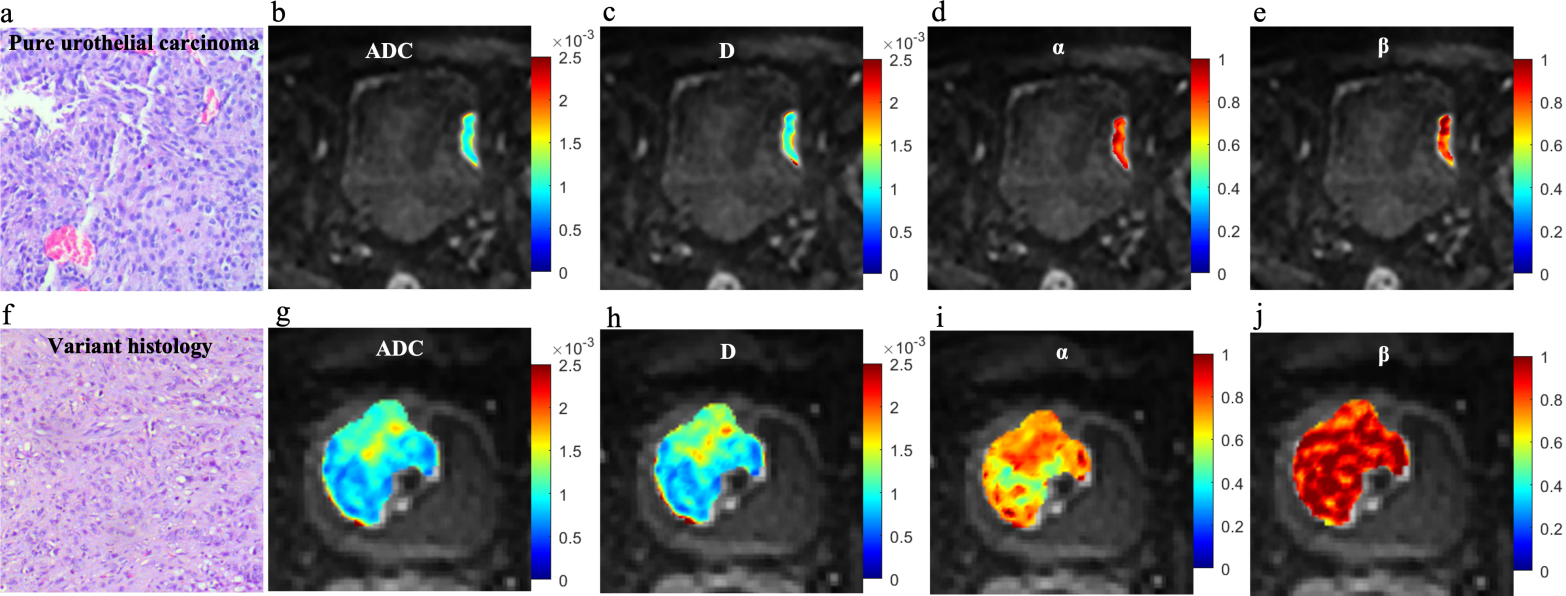
Microscopic pathology and colormaps of the apparent diffusion coefficient and continuous-time random-walk parameters of bladder tumors from a 71-year-old male with pure urothelial carcinoma (top row, **A-E**) and a 63-year-old male with urothelial carcinoma and sarcomatoid variant (bottom row, **F-J**). The urothelial carcinoma with variants showed lower ADC, D, and α values than pure urothelial carcinoma.

Significant decreases in ADC, D, and α values were observed in high-grade compared to low-grade BCa, as well as in VH relative to pUC. No significant difference in β was found between the low- and high-grade or between the pUC and VH (p = 0.808 and p = 0.059, respectively). Boxplots illustrating the comparison of ADC and CTRW parameters across different pathological groups are shown in **Figure 2**. Representative colormaps of ADC and CTRW parameters (D, α, and β) are shown in **Figures 3** and **4**. Parametric colormaps were smoothed via bilinear interpolation embedded in the ‘shading interp’ function in MATLAB.

### Performance of the diffusion parameters for pathological evaluation

**Figure 5:**
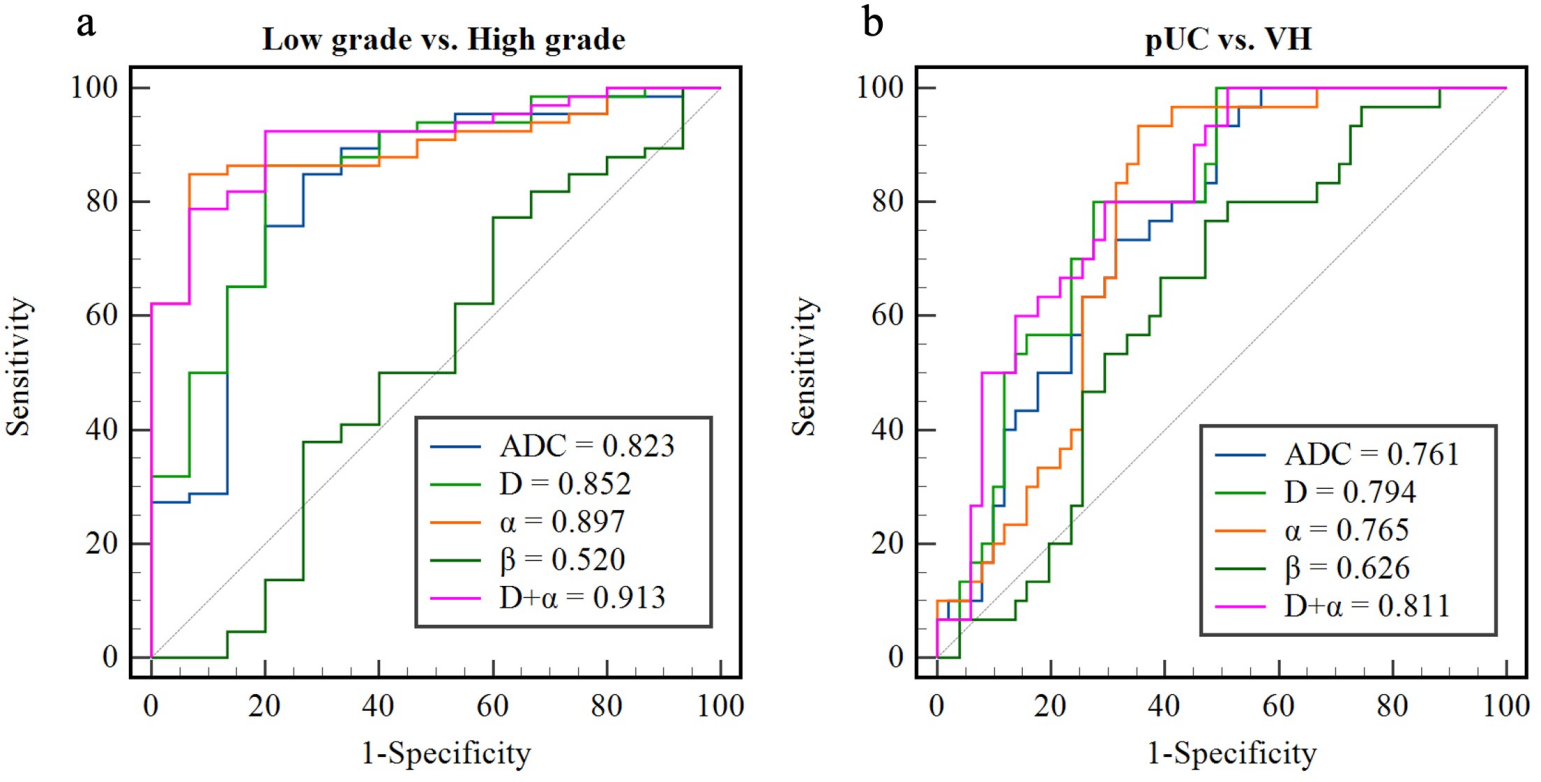
Receiver operating characteristic curves of the apparent diffusion coefficient, continuous-time random-walk parameters, and the combination of D and α for differentiating between low- and high-grade bladder cancer **(A)** and between pure urothelial carcinoma and variant histology **(B)**. pUC, pure urothelial carcinoma; VH, variant histology; ADC, apparent diffusion coefficient.

The diagnostic performance of ADC and CTRW parameters for differentiating between low- and high-grade BCa and between pUC and VH are listed in **Table 3**. The ROC curves for each parameter and their combinations are shown in **Figure 5**. Among the diffusion parameters for determining BCa grade, α exhibited the highest AUC (0.897), with D showing slightly higher AUC than ADC (0.852 vs. 0.823). However, no statistically significant differences were found in AUCs among α, D, and ADC (all p values > 0.05). In distinguishing VH from pUC, the highest AUC was found for D (0.794), which was significantly higher than that of ADC (0.761, p = 0.024). The α had similar AUC to ADC (0.765 vs. 0.761) with no significant difference between them (p = 0.951).

**Table 3:** Diagnostic performance of diffusion parameters for distinguishing different pathological group.

	ADC	D	α	β	CTRW (D+α)
Low vs. high grade					
AUC (95% CI)	0.823 (0.723-0.899)	0.852 (0.755-0.921)	0.897 (0.809-0.953)	0.520 (0.406-0.633)	0.913 (0.829-0.964)
Sensitivity (%)	84.85	86.36	84.85	77.27	92.42
Specificity (%)	73.33	80.00	93.33	40.00	80.00
Accuracy (%)	82.72	85.19	86.42	70.37	90.12
pUC vs. VH					
AUC (95% CI)	0.761 (0.654-0.849)	0.794 (0.690-0.876)	0.765 (0.657-0.852)	0.626 (0.512-0.731)	0.811 (0.709-0.890)
Sensitivity (%)	93.33	80.00	93.33	76.67	80.00
Specificity (%)	50.98	72.50	64.71	52.94	72.55
Accuracy (%)	66.67	75.31	75.31	61.73	75.31

ADC, apparent diffusion coefficient; AUC, area under the curve; CI, confidence interval; CTRW, continuous-time random-walk; pUC, pure urothelial carcinoma; VH, variant histology.

Univariate logistic regression analysis revealed that D and α were significantly associated with high grade and VH (p < 0.05). Multivariate logistic regression demonstrated that the combination of D and α (D+α) yielded significantly higher AUC than ADC for differentiation between low- and high-grade BCa (0.913 vs. 0.823, p = 0.043), and between pUC and VH (0.811 vs. 0.761, p = 0.026). For determining the pathological grade, the combination of D and α showed higher sensitivity (92.42% vs. 84.85%), higher specificity (80.00% vs. 73.33%), and higher accuracy (90.12% vs. 82.72%) compared to ADC. For the evaluation of VH, the specificity and accuracy of the combination of CTRW parameters were increased compared to ADC (72.55% vs. 50.98% and 75.31% vs. 66.67%), but the sensitivity was decreased (80.00% vs. 93.33%).

## Discussion

Our study demonstrated that both conventional ADC and CTRW parameters were effective in distinguishing between different pathological grades and variants in BCa. These diffusion parameters (ADC, D, and α) significantly differed between low- and high-grade BCa and between pUC and VH. Notably, the α parameter derived from the CTRW model showed the highest performance for BCa grading, while the D parameter from the CTRW model was most effective in differentiating VH from pUC. Furthermore, the combination of CTRW parameters (D+α) achieved a higher AUC than individual parameters, marking a significant improvement over the conventional ADC. These findings suggest that the CTRW model might be a potential tool for noninvasively investigating the pathological characteristics of BCa, offering a supplementary diagnostic approach to the current clinical reliance on TURBT.

Diffusion MRI stands as a powerful tool for probing biological microstructures and has shown potential as an imaging-based marker for predicting the pathological features of BCa (9, 31, 32). The mono-exponential model and its quantitative ADC parameter assume that water molecules follow Gaussian motion in a homogeneous medium. However, tissue structures, especially tumors, present heterogeneous and complex cellular and subcellular microstructures, influencing the diffusion of water molecules in a manner that diverges from a Gaussian distribution. Therefore, ADC may not be accurate enough to evaluate the heterogeneous pathological microstructure of BCa (17). Unlike the mono-exponential diffusion model, non-Gaussian diffusion models can reveal intravoxel tissue cellularity, the extracellular matrix, vascularity, and microstructures of tumors (33). The CTRW model provides insights into the intravoxel heterogeneity of water molecule diffusion both temporally (α) and spatially (β). Our study revealed that the CTRW model could not only aid in evaluating the pathological grade and variant of BCa but also exhibit superior diagnostic capabilities when compared to ADC, reinforcing its potential role in enhancing BCa diagnosis and treatment strategies.

The pathological condition plays a crucial role in guiding the management and forecasting of BCa outcomes (1). The determination of pathologic grade and histologic type typically relies on invasive TURBT procedures, but inadequate resection of the tumor may result in an inaccurate diagnosis (7). BCa grading is based on the organization of cytologic features and architectural abnormalities of the papillae (34). Low-grade BCa exhibits minimal variability in architecture and cytologic features. In contrast, high-grade BCa is characterized by a disorderly appearance resulting from marked architectural and cytologic abnormalities (35). The D parameter from the CTRW model describes the anomalous diffusion process and is analogous to ADC, which is related to tumor cellularity. Our results revealed significantly lower ADC and D values in high-grade compared to low-grade BCa, consistent with previous studies (10–13). This decrease can be attributed to the heightened tumor cellularity and decreased extracellular space of high-grade BCa, which consequently impedes water molecule diffusion (36). In addition, high-grade BCa showed increased tumor microstructure heterogeneity, with pronounced architectural and cytologic atypia (37). Our results showed that the temporal diffusion heterogeneity-sensitive parameter α surpassed other diffusion metrics in accurately distinguishing low- from high-grade BCa, indicating the utility of tumor heterogeneity as a grading parameter.

Bladder urothelial carcinoma is recognized for its morphological diversity, encompassing various histological subtypes and divergent differentiations (1). A careful morphological description of the presence of variants is crucial in BCa, as it may affect management and survival expectations (4). Mixed histological components reflect a more heterogeneous tumor microenvironment. Our study observed a lower α value in VH compared to pUC, reflecting the complex and heterogeneous histological components of VH. Additionally, the difference in ADC and D values may be attributed to the varying degrees of cellularity between pUC and VH. The D derived from CTRW presented the highest AUC in the evaluation of VH. The results of this study suggested that the quantitative parameters from the CTRW model could potentially aid pathologists in identifying concurrent histological variants with urothelial carcinoma.

Both α and β are indicative of diffusion heterogeneity within the tumor microstructure. The α parameter is associated with temporal diffusion heterogeneity, revealing the likelihood of water molecules being “retained” or “released” during diffusion, resulting in a variable time during each movement (22). Our findings showed lower α values in higher grades and VH, suggesting a more variable time for diffusing water molecules to travel through them and indicating greater temporal inhomogeneity. The β parameter is related to spatial diffusion heterogeneity and describes the different step lengths of water molecules during diffusion (23). Inconsistency was observed between the α and β values in this study, with no significant differences in the β values between low- and high-grade BCa or between pUC and VH. Water molecules can walk a variable spatial length during each move or spend a variable temporal interval to make a move. The discordant results between α and β in our study could be attributed to the variable time required by water molecules for movement in high-grade or VH BCa, without necessarily resulting in significantly varied displacements. Similarly, previous studies have reported inconsistent changes in α and β in other tumors (24, 38), suggesting that α and β reflect the heterogeneous microstructure in different aspects.

Compared to the single ADC, the CTRW model has the advantage of integrating multiple parameters. The CTRW model encapsulates the attributes of a non-Gaussian distribution and takes into consideration the underlying tumor tissue cellularity and heterogeneity, while ADC does not reveal intravoxel tissue heterogeneity according to the Gaussian distribution. In the present study, the combination of the CTRW parameters (D+α) resulted in the best performance compared with the individual parameters in grade and VH determination. Similarly, Du et al. reported that the combination of CTRW parameters yielded the highest AUC in the differentiation of benign and malignant breast lesions (21). Chang et al. reported that combining multiple CTRW parameters improved the performance of diagnosing molecular subtypes of breast cancer (39). These findings suggest that the combination of the CTRW parameters, which are related to tumor cellularity and heterogeneity, greatly improves the evaluation of pathological changes in tumors compared to individual parameters.

Most clinical MRI scanners allow the DWI signal to be acquired by varying only the diffusion gradient strength (g). Therefore, it is permissible to quantify only D and β by changing gradient strength because in this way D and β have a biophysical meaning. The current α is an estimated parameter extracted from a signal representation model and does not have a biophysical meaning (40). To quantify the true α that characterizes subdiffusion, it is essential to acquire DWI at different diffusion times (Δ). By varying the Δ value while keeping g constant, the decay of the DWI signal depends on Δ and α, where the α quantifies the true subdiffusion (41). In our study, the α does not quantify true subdiffusion because the data were acquired at a fixed diffusion time. Even though, the α parameter was a potential image marker and beneficial in determining pathological states, including grades and variants of BCa. Furthermore, the combination of α and D achieved the greatest discriminating power than individual diffusion parameters.

This study has several limitations. Firstly, the distribution of pathological grades was nonuniform, with more high-grade BCa than low-grade BCa and more pUC than VH, which may introduce bias to the statistical analysis. Secondly, a representative two-dimensional ROI was selected for diffusion parameter measurements, which may not fully reflect whole-tumor characteristics. A three-dimensional whole tumor volume analysis might offer more comprehensive information on BCa but would be more complex and time-consuming. Thirdly, the subgroups of different variants were not further statistically analyzed due to the limited sample size. Future studies with larger sample sizes are needed to validate the role of diffusion models in differentiating different subtypes of variants in BCa.

In conclusion, this study highlighted the distinctiveness of D and α from the non-Gaussian CTRW model and ADC from the mono-exponential model in distinguishing between low- and high-grade BCa as well as between pUC and VH. The CTRW model helped evaluate the grade and variant of BCa. Moreover, the combination of CTRW parameters representing tissue cellularity and heterogeneity outperformed the conventional ADC. Thus, the CTRW model could serve as a promising noninvasive tool, potentially complementing the current pathological evaluation relying on invasive TURBT.

## Data Availability

The datasets presented in this article are not readily available because the clinical and confidential nature of the material but can be made available from the corresponding author on reasonable request. Requests to access the datasets should be directed to JQ, qjx761225@126.com.

## References

[1] Humphrey PA Moch H Cubilla AL Ulbright TM Reuter VE. The 2016 WHO classification of tumours of the urinary system and male genital organs—Part B: prostate and bladder tumours. Eur Urol. (2016) 70:106–19. doi: 10.1016/j.eururo.2016.02.028 26996659

[2] National Comprehensive Cancer Network. (NCCN) clinical practice guidelines in oncology. Bladder cancer, version 1. 2024. Available online at: https://www.nccn.org/professionals/physician_gls/pdf/bladder.pdf (Accessed January 2024).

[3] Lopez-beltran A Cookson MS Guercio BJ Cheng L. Advances in diagnosis and treatment of bladder cancer. BMJ. (2024) 384:e076743. doi: 10.1136/bmj-2023-076743 38346808

[4] Black AJ Black PC. Variant histology in bladder cancer: Diagnostic and clinical implications. Transl Cancer Res. (2020) 9:6565–75. doi: 10.21037/tcr-20-2169 PMC8798576 35117266

[5] Klaile Y Schlack K Boegemann M Steinestel J Schrader AJ Krabbe LM. Variant histology in bladder cancer: How it should change the management in non-muscle invasive and muscle invasive disease? Transl Androl Urol. (2016) 5:692–701. doi: 10.21037/tau.2016.06.13 27785426 PMC5071184

[6] Deuker M Martin T Stolzenbach F Rosiello G Collà Ruvolo C Nocera L. Bladder cancer: A comparison between non-urothelial variant histology and urothelial carcinoma across all stages and treatment modalities. Clin Genitourin Cancer. (2021) 19:60–68.e1. doi: 10.1016/j.clgc.2020.07.011 32782133

[7] Babjuk M Burger M Capoun O Cohen D Compérat EM Dominguez Escrig JL. European association of urology guidelines on non–muscle-invasive bladder cancer (Ta, T1, and carcinoma in situ). Eur Urol. (2022) 81:75–94. doi: 10.1016/j.eururo.2021.08.010 34511303

[8] Hensley PJ Panebianco V Pietzak E Kutikov A Vikram R Galsky MD. Contemporary staging for muscle-invasive bladder cancer: accuracy and limitations. Eur Urol Oncol. (2022) 5:403–11. doi: 10.1016/j.euo.2022.04.008 35581143

[9] Yoshida S Takahara T Kwee TC Waseda Y Kobayashi S Fujii Y. DWI as an imaging biomarker for bladder cancer. Am J Roentgenol. (2017) 208:1218–28. doi: 10.2214/AJR.17.17798 28245143

[10] Takeuchi M Sasaki S Ito M Okada S Takahashi S Kawai T. Urinary bladder cancer: diffusion-weighted MR imaging–accuracy for diagnosing T stage and estimating histologic grade. Radiology. (2009) 251:112–21. doi: 10.1148/radiol.2511080873 19332849

[11] Wang Y Hu D Yu H Shen Y Tang H Kamel IR. Comparison of the diagnostic value of monoexponential, biexponential, and stretched exponential diffusion-weighted MRI in differentiating tumor stage and histological grade of bladder cancer. Acad Radiol. (2019) 26:239–46. doi: 10.1016/j.acra.2018.04.016 29753491

[12] Sevcenco S Ponhold L Heinz-Peer G Fajkovic H Haitel A Susani M. Prospective evaluation of diffusion-weighted MRI of the bladder as a biomarker for prediction of bladder cancer aggressiveness. Urol Oncol Semin Orig Investig. (2014) 32:1166–71. doi: 10.1016/j.urolonc.2014.04.019 24962659

[13] Yuan L Li D Mu D Zhang X Kong W Cheng L. Combined T2 SPAIR, dynamic enhancement and DW imaging reliably detect T staging and grading of bladder cancer with 3.0T MRI. Front Oncol. (2020) 10:582532. doi: 10.3389/fonc.2020.582532 33244456 PMC7683786

[14] Netto GJ Amin MB Berney DM Compérat EM Gill AJ Hartmann A. The 2022 world health organization classification of tumors of the urinary system and male genital organs—Part B: prostate and urinary tract tumors. Eur Urol. (2022) 82:469–82. doi: 10.1016/j.eururo.2022.07.002 35965208

[15] Lobo N Shariat SF Guo CC Fernandez MI Kassouf W Choudhury A. What is the significance of variant histology in urothelial carcinoma? Eur Urol Focus. (2020) 6:653–63. doi: 10.1016/j.euf.2019.09.003 31530497

[16] Alderson M Grivas P Milowsky MI Wobker SE. Histologic variants of urothelial carcinoma: morphology, molecular features and clinical implications. Bl Cancer. (2020) 6:107–22. doi: 10.3233/BLC-190257

[17] Lin WC Chen JH. Pitfalls and limitations of diffusion-weighted magnetic resonance imaging in the diagnosis of urinary bladder cancer. Transl Oncol. (2015) 8:217–30. doi: 10.1016/j.tranon.2015.04.003 PMC4487794 26055180

[18] Wang F Wu LM Hua XL Zhao ZZ Chen XX Xu JR. Intravoxel incoherent motion diffusion-weighted imaging in assessing bladder cancer invasiveness and cell proliferation. J Magn Reson Imaging. (2018) 47:1054–60. doi: 10.1002/jmri.25839 28815808

[19] Feng C Wang Y Dan G Zhong Z Karaman MM Li Z. Evaluation of a fractional-order calculus diffusion model and bi-parametric VI-RADS for staging and grading bladder urothelial carcinoma. Eur Radiol. (2022) 32:890–900. doi: 10.1007/s00330-021-08203-2 34342693

[20] Fokkinga E Hernandez-Tamames JA Ianus A Nilsson M Tax CMW Perez-Lopez R. Advanced diffusion-weighted MRI for cancer microstructure assessment in body imaging, and its relationship with histology. J Magn Reson Imaging. (2023) Published online. doi: 10.1002/jmri.29144 38032021

[21] Du M Zou D Gao P Yang Z Hou Y Zheng L. Evaluation of a continuous-time random-walk diffusion model for the differentiation of Malignant and benign breast lesions and its association with Ki-67 expression. NMR BioMed. (2023) 36:e4920. doi: 10.1002/nbm.4920 36912198

[22] Karaman MM Sui Y Wang H Magin RL Li Y Zhou XJ. Differentiating low- and high-grade pediatric brain tumors using a continuous-time random-walk diffusion model at high b-values. Magn Reson Med. (2016) 76:1149–57. doi: 10.1002/mrm.26012 PMC4852163 26519663

[23] Karaman MM Zhang J Xie KL Zhu W Zhou XJ. Quartile histogram assessment of glioma Malignancy using high b-value diffusion MRI with a continuous-time random-walk model. NMR BioMed. (2021) 34:1–13. doi: 10.1002/nbm.4485 33543512

[24] Li C Wen Y Xie J Chen Q Dang Y Zhang H. Preoperative prediction of VETC in hepatocellular carcinoma using non-Gaussian diffusion-weighted imaging at high b values: a pilot study. Front Oncol. (2023) 13:1167209. doi: 10.3389/fonc.2023.1167209 37305565 PMC10248416

[25] Tang L Zhou XJ. Diffusion MRI of cancer: From low to high b-values. J Magn Reson Imaging. (2019) 49:23–40. doi: 10.1002/jmri.26293 30311988 PMC6298843

[26] Kobayashi S Koga F Kajino K Yoshita S Ishii C Tanaka H. Apparent diffusion coefficient value reflects invasive and proliferative potential of bladder cancer. J Magn Reson Imaging. (2014) 39:172–8. doi: 10.1002/jmri.24148 23589321

[27] Panebianco V Narumi Y Altun E Bochner BH Efstathiou JA Hafeez S. Multiparametric magnetic resonance imaging for bladder cancer: development of VI-RADS (Vesical imaging-reporting and data system). Eur Urol. (2018) 74:294–306. doi: 10.1016/j.eururo.2018.04.029 29755006 PMC6690492

[28] Ingo C Magin RL Colon-Perez L Triplett W Mareci TH. On random walks and entropy in diffusion-weighted magnetic resonance imaging studies of neural tissue. Magn Reson Med. (2014) 71:617–27. doi: 10.1002/mrm.24706 PMC4930657 23508765

[29] Garrappa R. Numerical evaluation of two and three parameter Mittag-Leffler functions. SIAM J Numer Anal. (2015) 53:1350–69. doi: 10.1137/140971191

[30] Padhani AR Liu G Mu-Koh D Chenevert TL Thoeny HC Takahara T. Diffusion-weighted magnetic resonance imaging as a cancer biomarker: Consensus and recommendations. Neoplasia. (2009) 11:102–25. doi: 10.1593/neo.81328 PMC2631136 19186405

[31] van der Pol CB Chung A Lim C Gandhi N Tu W McInnes MDF. Update on multiparametric MRI of urinary bladder cancer. J Magn Reson Imaging. (2018) 48:882–96. doi: 10.1002/jmri.26294 30221801

[32] De Perrot T Zoua CS Glessgen CG Botsikas D Berchtold L Salomir R. Diffusion-weighted MRI in the genitourinary system. J Clin Med. (2022) 11:1–23. doi: 10.3390/jcm11071921 PMC9000195 35407528

[33] Tang C Li F He L Hu Q Qin Y Yan X. Comparison of continuous-time random walk and fractional order calculus models in characterizing breast lesions using histogram analysis. Magn Reson Imaging. (2024) 108:47–58. doi: 10.1016/j.mri.2024.01.012 38307375

[34] Wang G McKenney JK. Urinary bladder pathology: World Health Organization classification and American joint committee on cancer staging update. Arch Pathol Lab Med. (2019) 143:571–7. doi: 10.5858/arpa.2017-0539-RA 30044124

[35] Kirkali Z Chan T Manoharan M Algaba F Busch C Cheng L. Bladder cancer: Epidemiology, staging and grading, and diagnosis. Urology. (2005) 66:4–34. doi: 10.1016/j.urology.2005.07.062 16399414

[36] Hafeez S Huddart R. Advances in bladder cancer imaging. BMC Med. (2013) 11:104. doi: 10.1186/1741-7015-11-104 23574966 PMC3635890

[37] Álvarez KJ Campos HJP López BA Requena TMJ. The 2004 WHO classification of bladder tumors: A summary and commentary. Actas Urol Esp. (2007) 31:978–88. doi: 10.1016/s0210-4806(07)73761-0 18257367

[38] Mao C Hu L Jiang W Qiu Y Yang Z Liu Y. Discrimination between human epidermal growth factor receptor 2 (HER2)-low-expressing and HER2-overexpressing breast cancers: a comparative study of four MRI diffusion models. Eur Radiol. (2024) 34:2546–59. doi: 10.1007/s00330-023-10198-x 37672055

[39] Chang H Wang D Li Y Xiang S Yang YX Kong P. Evaluation of breast cancer Malignancy, prognostic factors and molecular subtypes using a continuous-time random-walk MR diffusion model. Eur J Radiol. (2023) 166:111003. doi: 10.1016/j.ejrad.2023.111003 37506477

[40] Novikov DS Kiselev VG Jespersen SN. On modeling. Magn Reson Med. (2018) 79:3172–93. doi: 10.1002/mrm.27101 PMC5905348 29493816

[41] Capuani S Palombo M. Mini review on anomalous diffusion by MRI: potential advantages, pitfalls, limitations, nomenclature, and correct interpretation of literature. Front Phys. (2020) 7:248. doi: 10.3389/fphy.2019.00248

